# Anatomical Behaviour of the Phrenic Nerve and Innervation of the Diaphragm in the Maned Wolf ( *Chrysocyon brachyurus* )

**DOI:** 10.1111/ahe.70087

**Published:** 2026-01-28

**Authors:** Beatriz Costa do Nascimento, Amanda Rohrs de Cerqueira, Gabriela Santana dos Anjos, Ingrid Bulhões Pimenta, Estela Larissa Silva dos Santos, Alberto Vinicius Dantas Oliveira, Paula Velozo Leal, Ricardo Diniz Guerra e Silva, Marcia Maria Magalhães Dantas de Faria, William Pérez, Érica Augusta dos Anjos Cerqueira‐Silva

**Affiliations:** ^1^ School of Veterinary Medicine and Zootechny, Federal University of Bahia Salvador Brazil; ^2^ Instituto do Meio Ambiente e Recursos Hídricos (INEMA Institute for the Environment and Water Resources) Bahia Brazil; ^3^ Pedeciba Universidad de Montevideo Montevideo Uruguay

**Keywords:** comparative neuroanatomy, peripheral nervous system, wild canid morphology

## Abstract

The phrenic nerve is responsible for the motor innervation of the diaphragm, which is essential for breathing. The literature lacks data regarding the nervous and respiratory systems of wild animals, especially the maned wolf ( *Chrysocyon brachyurus* ). Therefore, this study aimed to describe the origin and terminal distribution of the phrenic nerve in the diaphragm of *Chrysocyon brachyurus* . Seven maned wolf specimens were preserved by a 10% formaldehyde solution and were later dissected. The phrenic nerve most frequently originated from the ventral branches of C5, C6, and C7, forming trunks that merged when they reached the first rib. Upon the diaphragm, the phrenic nerve exhibited a terminal bifurcation into the lumbocostal trunk and the sternal branch. The distribution of the branches was symmetrical and specific to the lumbar, costal, and sternal portions of the diaphragm, with no innervation of the central tendon or caudal vena cava. It was not observed contributions from the intercostal nerves to the diaphragm innervation. It is concluded that the phrenic nerve of the maned wolf originates from the ventral branches of C5, C6, and C7, showing unisegmental or plurisegmental characteristics and representing the sole nervous supply to the diaphragm in this species. The findings demonstrate similarities with what is described in the crab‐eating fox, but there are significant differences compared to the domestic dog, indicating interspecies variability in phrenic nerve morphology among carnivores and highlighting the need for further comparative studies.

## 1 Introduction

The maned wolf ( *Chrysocyon brachyurus* ) is the largest South American canid, distinguished by its long legs and reddish coat. Found mainly in Brazil, Paraguay, Bolivia, and northern Argentina, it inhabits grasslands and savannas (Dietz 1984; Rodrigues et al. 2018). This solitary species is omnivorous, feeding on small animals and fruits—especially *Solanum lycocarpum* , which aids in seed dispersal (Motta‐Junior et al. 1996). The IUCN (2025) classifies it as Near Threatened due to habitat loss and human pressures. In this context, detailed anatomical studies provide valuable information for clinical, anaesthetic, surgical, and rehabilitative practices, as well as contributing to the knowledge of Brazilian biodiversity.

Understanding the anatomical characteristics of the peripheral nervous system in wild animals represents an essential tool for not only the advancement of veterinary medicine in itself, but also for species conservation. The phrenic nerve is a branch of the cervical plexus responsible for the diaphragm's motor innervation; therefore, it is essential for respiratory function. Its origin, course, and distribution have been extensively documented in domestic species such as dogs, cats, and cattle (Dyce et al. 2018; König and Liebich 2021). However, studies on wild animals, particularly species that are native to Brazil, such as the maned wolf, remain scarce.

Considering the functional importance of the phrenic nerve and the need to expand the knowledge about the anatomical aspects of endangered species, this study aims to describe the origin and distribution of the phrenic nerve in the diaphragm of *Chrysocyon brachyurus* , contributing to the comparative anatomy field, to future clinical applications, and to species conservation.

## 2 Materials and Methods

Seven male cadavers of *Chrysocyon brachyurus* of different ages were donated by the Instituto do Meio Ambiente e Recursos Hídricos (INEMA) to the Veterinary Anatomy Sector (SAV) – EMEVZ at the Federal University of Bahia. All procedures followed the guidelines of the Ethics Committee on Animal Use (CEUA, protocol no. 89/2023) and were authorised by the Brazilian Institute of Environment and Renewable Natural Resources (IBAMA), through the Biodiversity Authorization and Information System (SISBIO, permit no. 82780‐1).

To preserve the cadavers, a 10% formaldehyde solution was perfused through their common carotid artery, and later the specimens were submerged in a similar solution for 30 days. To access the phrenic nerve, a median incision was made in the ventral cervical skin, from the mandibular symphysis to the umbilical scar, with dorsal reflection. The first to the seventh pair of ribs and the lungs were removed. The dissection proceeded cranially to identify the ventral branches of the cervical spinal nerves that originate the phrenic nerve.

The diaphragm was exposed in its thoracic surface for the dissection of the phrenic nerve branches. The diaphragm was then removed after its lumbar insertion was dissected to verify any contribution from the lumbar branches. The intercostal nerves were also dissected to investigate potential contributions to the diaphragm's innervation. After proper identification, the structures were painted yellow with fabric dye, described, and photographed. Anatomical terminology followed the International Committee on Veterinary Gross Anatomical Nomenclature (International Committee on Veterinary Gross Anatomical Nomenclature (2017)) and Silva et al. (2022).

A descriptive statistical analysis was performed based on the frequency of the variables related to the branches that compose the phrenic nerve and their distribution in the diaphragm. To compare the right and left antimeres, Fisher's exact test was applied, as it is appropriate for the small sample size and the observed frequencies. Statistical analyses were performed using GraphPad Prism 10.0 (GraphPad Software, USA), and a significance level of *p* < 0.05 was adopted.

## 3 Results

The analysis of 14 cervical antimeres from *Chrysocyon brachyurus* (LG1–LG7) revealed that the phrenic nerve originated from different combinations of the ventral branches of cervical spinal nerves C5, C6, and C7. This pattern was observed in 57.1% of the cases on the right side and 83.3% on the left side. These ventral branches consistently merged when they reached the first rib. No significant difference in the distribution of the contributing roots (*p* = 0.3712), indicating that phrenic nerve formation is symmetrically distributed and suggesting a conserved anatomical pattern in this species. Overall, 64.3% (9/14) of the nerves exhibited the classical C5–C6–C7 formation.

Although this pattern was predominant, did not demonstrate statistical significance (*p* = 0,3712), likely due to the limited sample size. Nevertheless, the observed trend suggests that, with a larger sample size, the C5–C6–C7 configuration would likely be confirmed as statistically predominant.

**FIGURE 1:**
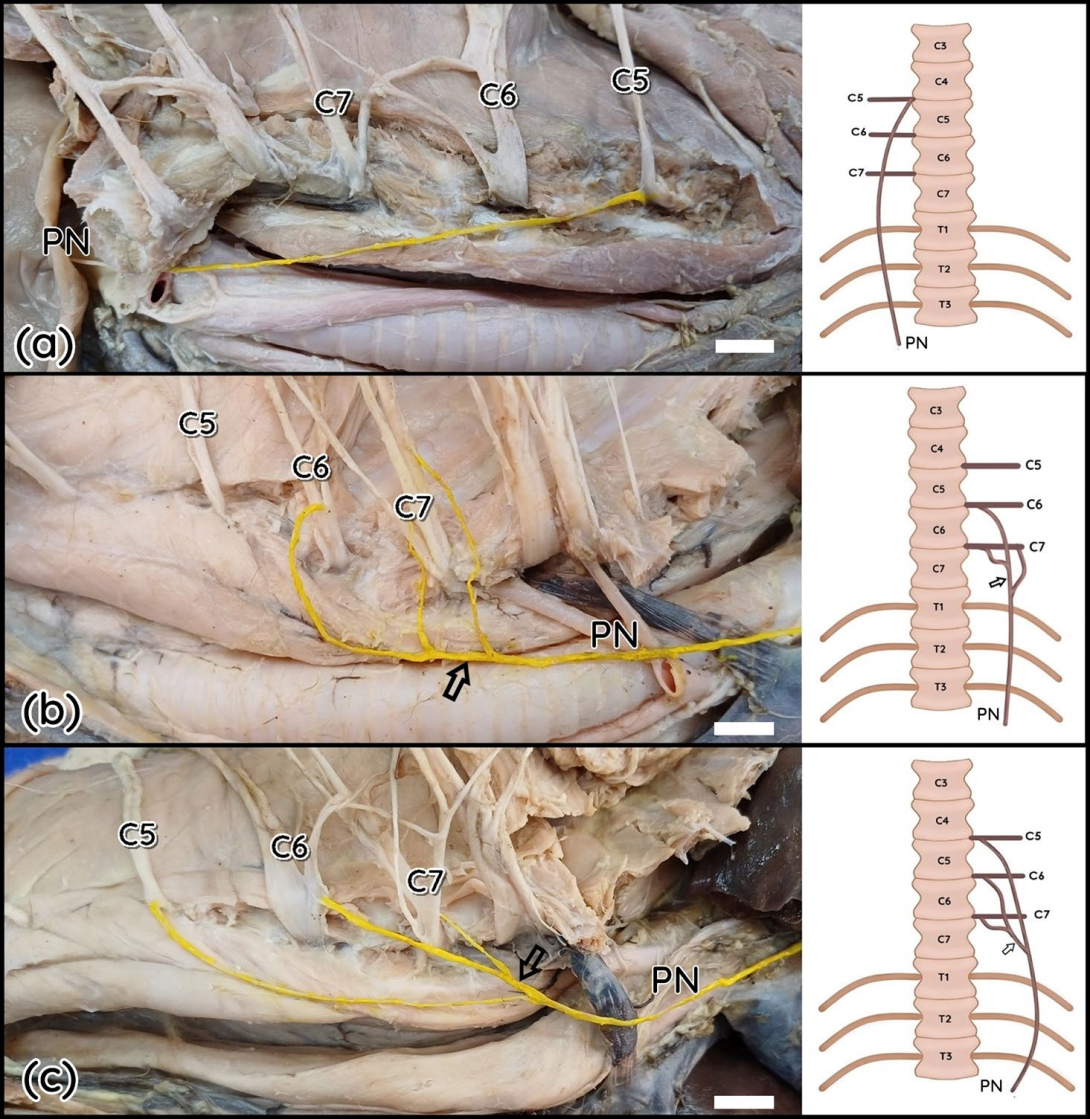
Photographs and schematic drawings of the cervical region of the maned wolf. Schematic drawings in ventral view. Right lateral view (a). Left lateral view (b and c). C5–C7 (fifth to seventh cervical spinal nerves); Phrenic nerve (PN). Note the formation of the C6–C7 trunk (hollow arrow). Bar scale 1 cm.

**FIGURE 2:**
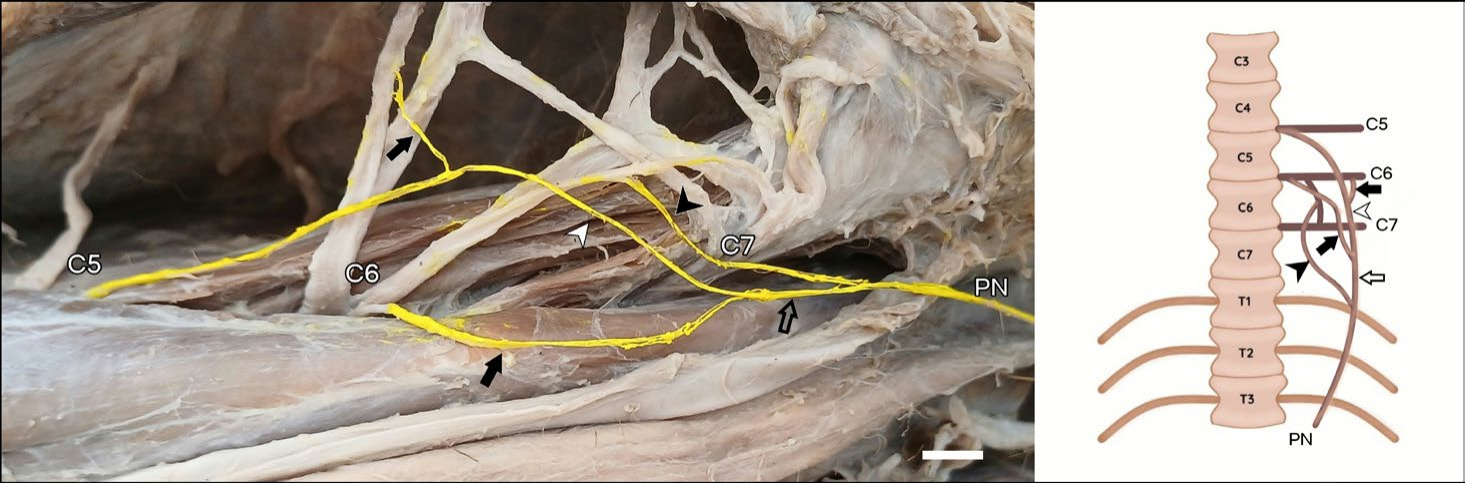
Photograph and schematic drawing of the cervical region of the maned wolf (LG7). Schematic drawings in ventral view. Left lateral view. The C6 nerve emits three branches contributing to the formation of the phrenic nerve (white arrow, black arrow, and black arrowhead). Trunk C5–C6 (white arrowhead); trunk C5 + C6–C6 (red arrowhead); branch from the C6–C7 connection (black arrowhead). Phrenic nerve (PN). Bar scale 1 cm.

In LG2, the right phrenic nerve originated exclusively from the ventral branch of C5 (Figure 1A). In LG7, the right antimere originated from two branches, C5 and C6. In other specimens (LG4, LG5, LG6), as well as in the left antimeres of LG1 and LG2 and in the right antimere of LG3, the classical triple origin pattern was observed, in which C5, C6, and C7 contribute to the phrenic nerve with one branch each (Figure 1C). The left phrenic nerve of LG1 was composed of one branch from C6 and two from C7 (Figure 1B). In LG7, the left antimere showed three branches from C6 (Figure 2) to the phrenic nerve. In LG3, the left phrenic nerve was originated by the union of one branch from C5 with the branch from the anastomotic trunk between C6 and C7, plus an additional branch from C7 (Figure 2).

**FIGURE 3:**
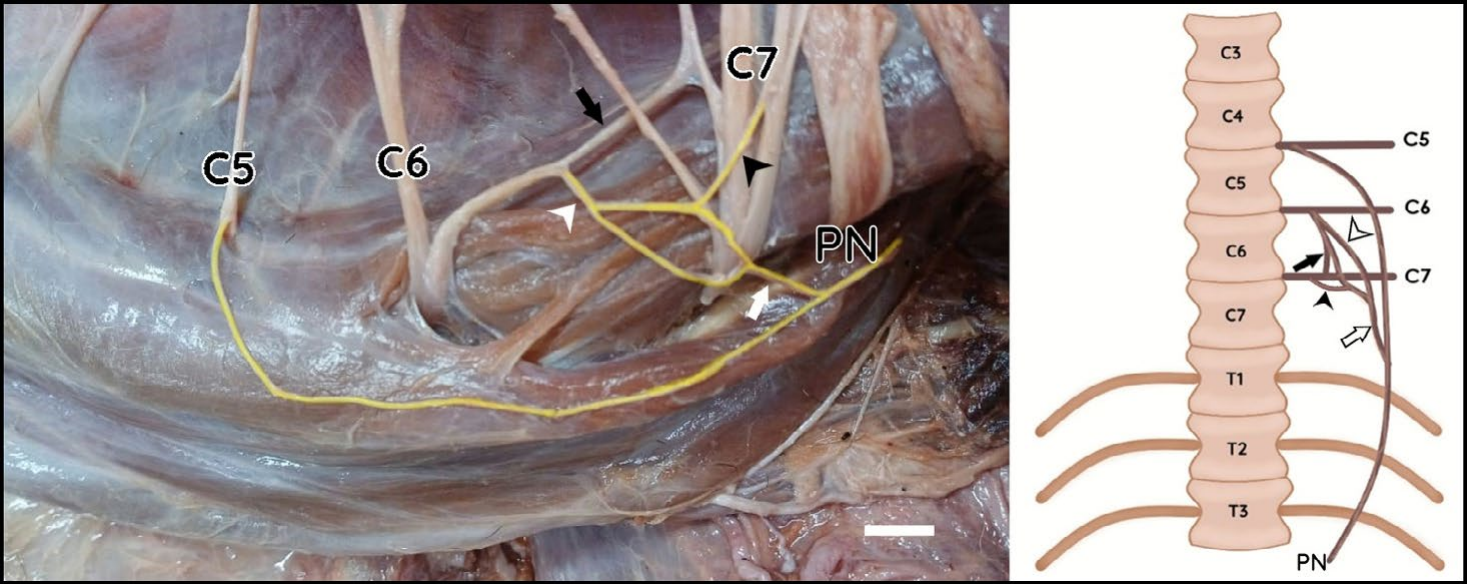
Photograph and schematic drawing of the cervical region of the maned wolf. Schematic drawings in ventral view. Left lateral view. Anastomotic branch between C6 and C7 (black arrow). Branch from C6 (white arrowhead); branch from C7 (black arrowhead); C6–C7 trunk (white arrow). Bar scale 1 cm.

**FIGURE 4:**
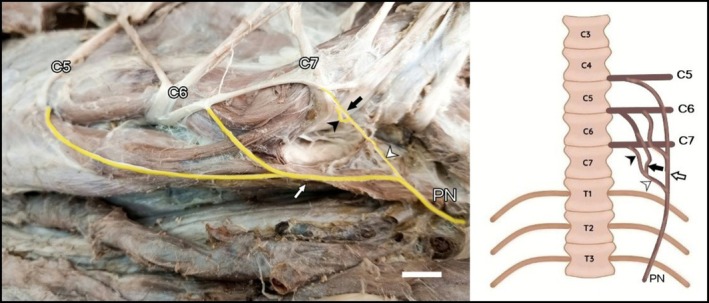
Photograph and schematic drawing of the cervical region of the maned wolf. Schematic drawings in ventral view. Ventral branches from C5 and C6 forming the C5–C6 trunk (white arrow). The black arrow indicates the branch from the C6–C7 anastomotic trunk joining the branch from C7 (black arrowhead), forming the C6–C7 trunk (white arrowhead). Bar scale 1 cm.

Anastomotic branches between C6 and C7 were observed in three specimens (LG3, LG5, and LG8). In those cases, the C6–C7 trunk emitted a branch that joined the C5–C6 trunk, collectively originating the phrenic nerve (Figures 3 and 4).

Regarding the number of forming branches, the classification of Almeida et al. (2008) was adopted: Type I (one branch), Type II (two branches), and Type III (three branches). The predominant configuration in this sample was Type III (Table 1).

**TABLE 1:** Origin of the branches forming the phrenic nerve in the maned wolf ( *Chrysocyon brachyurus* ).

Branches forming the phrenic nerve	Right phrenic nerve *n* (%)	Left phrenic nerve *n* (%)	Type
C5	1 (14.3%)	0 (0%)	I
C6 e C7	1 (14.3%)	1 (14.3%)	II
C5 e C7	0 (0%)	0 (0%)	II
C5 e C6	1 (14.3%)	1 (14.3%)	II
C5, C6 e C7	4 (57.1%)	5 (71.4%)	III
Total	7 (100%)	7 (100%)	—

**FIGURE 5:**
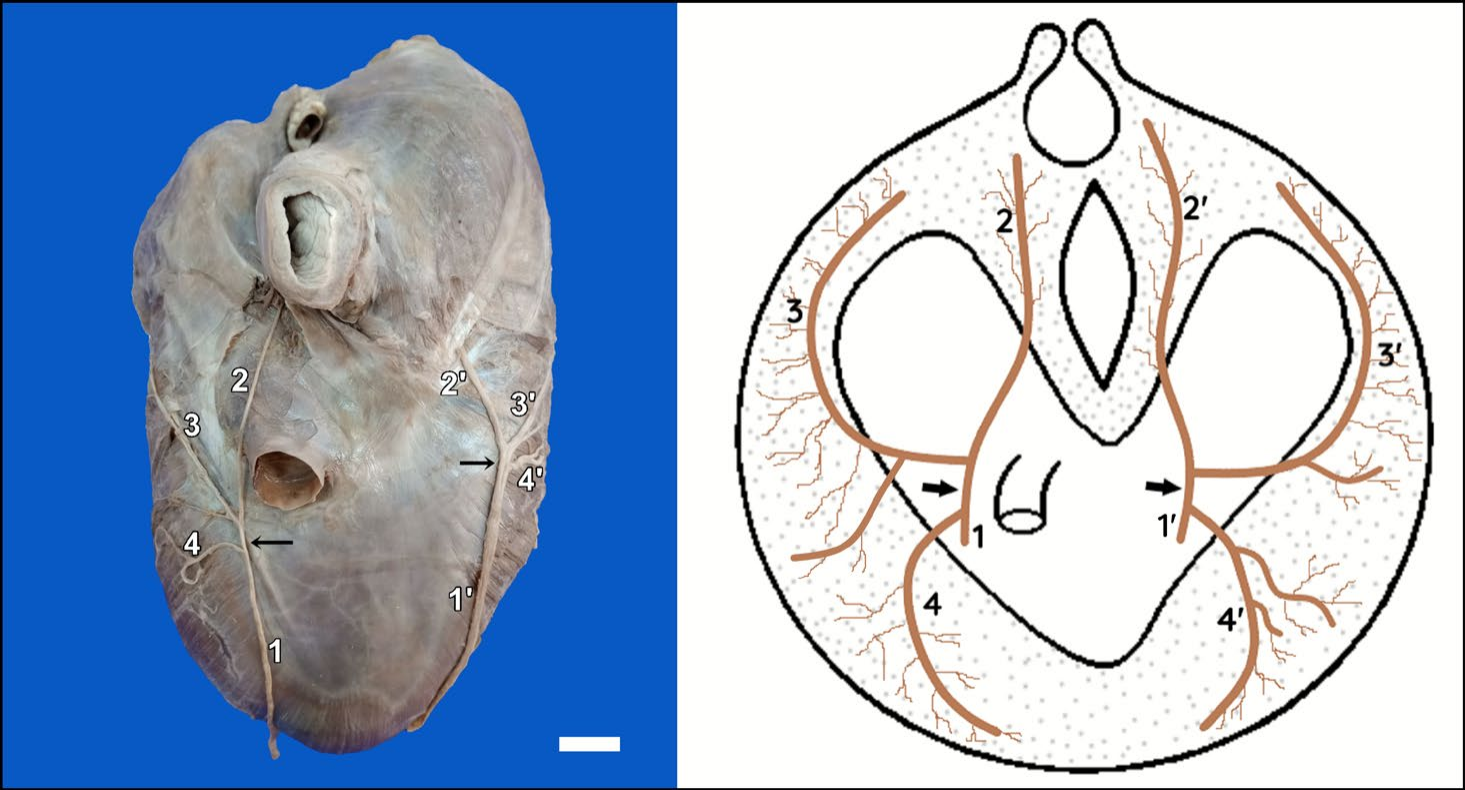
Distribution of the phrenic nerve on the thoracic surface of the diaphragm of the maned wolf. Lumbocostal trunk (black arrow); Phrenic nerve (1 and 1′); lumbar branch (2 and 2′); costal branch (3 and 3′); sternal branch (4 and 4′).

Upon reaching the diaphragm, both right and left phrenic nerves consistently bifurcated into a lumbocostal trunk, which originated the lumbar and costal branches, and a sternal branch (14/14; 100%) (Figure 5). The lumbar branch innervated the crura and the lumbar region of the diaphragm; the costal branch bifurcated and extended to the costal portion; and the sternal branch bifurcated and supplied the ventrolateral region of the costal part and the sternal part of the diaphragm. No branches directed to the central tendon or to the caudal vena cava were identified, and no fibre crossings or connections between ipsilateral or contralateral branches were observed (Figure 5).

## 4 Discussion

In the maned wolf specimens analysed, the phrenic nerve presented variable origins between spinal segments C5–C7, being formed by one (C5), two (C5 and C6 or C6 and C7), or three branches (C5, C6, C7), thus characterising unisegmental or plurisegmental nerves (Prada 2014). This configuration partially corresponds to that observed in domestic dogs and the crab‐eating fox, in which respectively Type II (C5 and C6) and Type III (C5, C6, and C7) predominated, with no records of Type I or Type II composed of C6–C7 (Silva et al. 2022).

The shared origin of the phrenic nerve with the brachial plexus (Souza Junior et al. 2014) has important clinical and anaesthetic implications. Because the phrenic nerve may arise from C5 to C7, regional anaesthetic techniques targeting the brachial plexus—particularly interscalene or supraclavicular blocks—may inadvertently affect the phrenic nerve, resulting in transient diaphragmatic paralysis. This is especially relevant in individuals with preexisting respiratory compromise. Furthermore, the contribution from C7 indicates that the phrenic nerve can be positioned more caudally than expected, requiring caution during cervical dissections or surgical approaches to the brachial plexus to prevent iatrogenic injury and associated respiratory dysfunction.

Type I has been reported in Santa Inês sheep, originating from C6 (Almeida et al. 2008), and in olive baboons ( *Papio anubis* ), originating from C4 (Champneys 1871). Type II has been described in brown‐throated sloths, originating from C8 and C9 (Nascimento 2018).

In maned wolves, Type III configuration was the most frequent, similar to that observed in the crab‐eating fox (Silva et al. 2022), but differing from the domestic dog, in which Type II (C5 and C6) predominated, and from the collared peccary ( *Tayassu tajacu* ), where Type IV (C4, C5, C6, and C7) was most frequent (Silva et al. 2025).

Variability in the origins and classification of the phrenic nerve across species, both domestic and wild, is well documented (Cassel et al. 2002), though no clear justification has been established. This corroborates Pancrazi's (1925) hypothesis that the origin and division of the phrenic nerve occur randomly, with variations even among individuals of the same species.

In maned wolves, the originating branches of the phrenic nerve converged into trunks when they reached the first rib, a pattern similar to that described in the crab‐eating fox (Silva et al. 2022) and in sheep (Almeida et al. 2008). This differs from the domestic dog, in which the branches follow independent courses before merging (Silva et al. 2022).

In all specimens analysed, the phrenic nerve bifurcated upon reaching the phrenic center, forming a lumbocostal trunk that originated the lumbar and costal branches, in addition to the sternal branch. This terminal branching was symmetrical between antimeres and coincides with patterns observed in other species, including the crab‐eating fox, domestic dog (Silva et al. 2022), domestic cat (Moreira et al. 2007), white‐eared opossum (Cassel et al. 2002), sheep (Almeida et al. 2008), pigs (Santos et al. 2019), and donkeys (Amorim Júnior et al. 1996).

Unlike sheep and marmosets (Almeida et al. 2008; Amorim Júnior et al. 1993), no trifurcated branching was observed in the maned wolf. The bifurcation consistently generated systematically distributed branches: the lumbar branch innervated only the lumbar portion, the costal branch the costal portion, and the sternal branch both the sternal and costal portions simultaneously.

This pattern is consistent with that observed in the crab‐eating fox (Silva et al. 2022) and partially consistent with the domestic cat, in which overlapping areas of innervation were reported (Moreira et al. 2007), as well as in the giant anteater (Silva et al. 2022).

No branches directed to the central tendon or caudal vena cava were identified in the specimens analysed, a finding consistent with domestic dogs, crab‐eating foxes (Silva et al. 2022), and cats (Moreira et al. 2007), but differing from opossums (Cassel et al. 2002), giant anteaters (Silva et al. 2022), and collared peccaries (Silva et al. 2025), in which these structures received additional innervation.

Although the literature reports occasional contributions from intercostal nerves to the diaphragmatic innervation in domestic dogs (Dyce et al. 2018), cats (Faria et al. 2011), sheep (Almeida et al. 2008), guinea pigs (Faria et al. 2019), and primates (Rosenblueth et al. 1961), such participation was not observed in the specimens analysed in the present study, similar to what has been described in the crab‐eating fox ( *Cerdocyon thous* ) (Silva et al. 2022). This absence reinforces the individual variability of diaphragmatic innervation patterns and suggests that the phrenic nerve alone may provide complete motor supply in some individuals.

From a clinical and surgical perspective, knowledge of this branching pattern is essential during thoracic or diaphragmatic interventions to avoid inadvertent nerve damage, which could compromise respiratory function. Furthermore, the absence of intercostal contributions reinforces the importance of preserving the phrenic nerve during procedures, as it represents the sole motor supply to the diaphragm in this species.

## 5 Conclusion

It is concluded that the phrenic nerve of the maned wolf ( *Chrysocyon brachyurus* ) originates predominantly from the ventral branches of cervical spinal nerves C5, C6, and C7, being classified as Type III, with plurisegmental characteristics. Less frequently, Type II and, rarely, Type I configurations were observed. Upon reaching the diaphragm, the phrenic nerves bifurcated into a lumbocostal trunk—that in its turn originated the lumbar and costal branches—and a sternal branch, thus representing the exclusive motor innervation of the diaphragm. This morphological pattern resembles that observed in the crab‐eating fox ( *Cerdocyon thous* ), reinforcing possible anatomical affinities among these wild canids.

## Funding

This study was supported by the Programa de Apoio a Jovens Professores(as)/Pesquisadores(as) Doutores(as) (JOVEMPESQ), an institutional research funding program of the Universidade Federal da Bahia (UFBA). The financial resources are provided by UFBA and administered by the Pró‐Reitoria de Pesquisa e Pós‐Graduação (PRPPG/UFBA), which manages the JOVEMPESQ program. No external funding agency was involved.

## Conflicts of Interest

The authors declare no conflicts of interest.

## Data Availability

The data that support the findings of this study are available from the corresponding author upon reasonable request.
